
JOURNAL INFORMATION
==============================
NLM Title Abbreviation: Insights Imaging
Journal ID: Insights Imaging
NLM Journal ID: 101532453

Insights into Imaging

EISSN: 1869-4101
Publisher: Springer

ARTICLE INFORMATION
==============================
PMCID: PMC7046845
PMID: 32107668
DOI: 10.1186/s13244-020-00849-8
Article ID: 849
Article version: 1
Subjects: Meeting Abstracts

EuSoMII Annual Meeting 2019 Book of abstracts

Electronic publication date: 2020 Feb 28
Publication date: 2020 Feb
Volume: 11
Issue: Suppl 2
Issue sponsor: EuSoMII 2019 is the annual meeting of the European Society of Medical Imaging Informatics. Publication costs were not supported by sponsorship.
Electronic Location ID: 25
Copyright: © The Author(s) 2020
License: Open AccessThis article is distributed under the terms of the Creative Commons Attribution 4.0 International License (http://creativecommons.org/licenses/by/4.0/), which permits unrestricted use, distribution, and reproduction in any medium, provided you give appropriate credit to the original author(s) and the source, provide a link to the Creative Commons license, and indicate if changes were made.
License URL: https://creativecommons.org/licenses/by/4.0/

Conference name: EuSoMII Annual Meeting 2019 Medical Imaging Informatics - AI, Clinical Applications and more
Conference location: Valencia, Spain
Conference date: 18-19 October 2019
issue-copyright-statement: © The Author(s) 2020

==============================
EuSoMII

### Insights into Imaging

Education and strategies in European radiology

EuSoMII Annual Meeting 2019 Book of abstracts / Volume 11 / Supplement 2

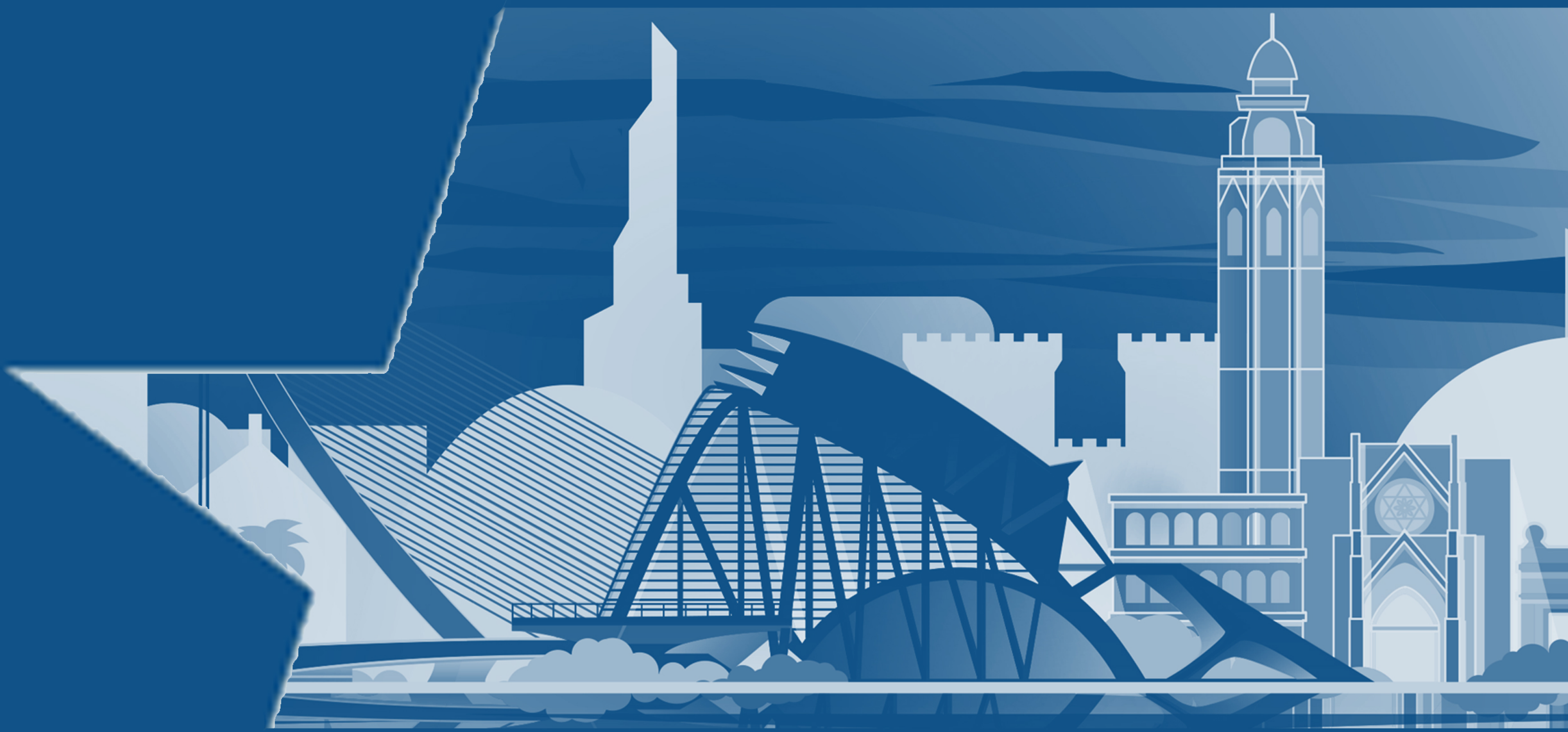

EuSoMII Annual Meeting 2019
Medical Imaging Informatics -
AI, Clinical Applications and more
Valencia, October 18-19, 2019

ESR
EUROPEAN SOCIETY OF RADIOLOGY

### EuSoMII

### Annual Meeting

Medical Imaging Informatics
AI, Clinical Applications and more

Valencia
October 18 - 19, 2019

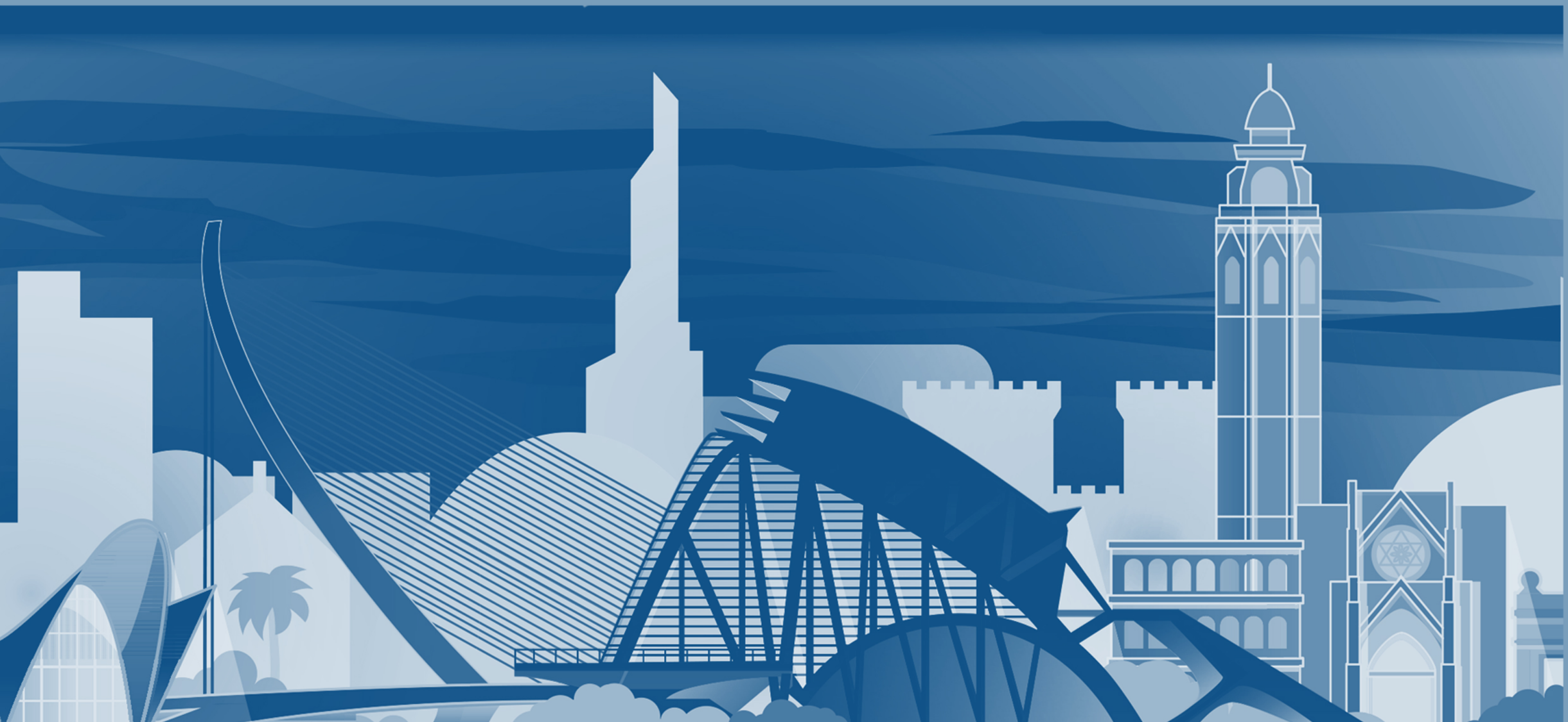

### EUSOMII
### ANNUAL MEETING 2019
### SCIENTIFIC
### SESSIONS

Insights Imaging (2020) 11 (Suppl 2): 25
https://doi.org/10.1186/s13244-020-00849-8
Published Online: 28 February 2020

### SS 1

### Evaluation of the Use of Convolutional Neural Networks with Variable Stride for Skin Lesion Classification

*Oge Marques, PhD and Luiz Zaniolo, MS; Florida Atlantic University*

Short Summary: The convolution layers of a Convolutional Neural Network (CNN) include a *stride* parameter that dictates how big are the steps for sampling when scanning the input layer to run convolutional operations. The vast majority of CNNs use a fixed stride value. This work reports experiments designed to test in a systematic way the following hypothesis: "The use of variable stride in skin lesion images will lead to improved performance (when compared to the baseline case of comparable computational complexity, i.e., fixed stride = 2)."

Purpose/Objectives: The purpose of this work is to demonstrate that by changing the stride value in CNNs depending on the position of the pixel within the image (smaller stride for the center of the image, and larger one for pixels close to the edges), an increase in processing speed in both training and recognition phases can be achieved without sacrificing accuracy.

Methods and materials: The network used for the experiments follows a typical CNN architecture for image classification tasks, whose layers include: one block of Convolutional 2D (the layer in which we modify the *stride* parameter) + Batch Normalization + ReLU + Max Pooling layers; two blocks of Convolutional 2D + Batch Normalization + ReLU layers (with standard stride 1); a Fully connected layer, a Softmax layer, and a Classification layer.

We used the HAM10000 dataset, which consists of a large collection of dermatoscopic images, divided into seven different classes: Melanocytic nevi, Melanoma, Benign keratosis-like lesions, Basal cell carcinoma, Actinic keratoses, Vascular lesions, and Dermatofibroma. 95% of the images were randomly selected for training and 5% used for validation.

Experiments were performed in four different network configurations: fixed stride of (1, 2, or 3) or variable stride. We chose accuracy as a metric of classification performance and also measured training time and inference time.

Results: Experimental results (Table 1) have confirmed our hypothesis. In this case, the accuracy for the variable stride case was not only higher than the baseline case (stride 2) but also the highest overall.

| Stride | Accuracy | Training Time | Inference Time |
|---|---|---|---|
| 1 | 77.0 % | 258 min | 31.9 ms |
| 2 | 76.4 % | 105 min | 10.9 ms |
| 3 | 75.5 % | 70 min | 5.8 ms |
| **Variable** | **78.1 %** | **105 min** | **10.9 ms** |

Table 1: Skin lesion classification using the HAM10000 dataset and variable stride.

Conclusion: We evaluated a method for using CNNs with variable stride for skin lesion classification and demonstrated that they can achieve higher accuracy and take less time to train or make predictions than the same networks using fixed stride.

Disclosure: No disclosures.

Keywords: Deep Learning; Convolutional Neural Networks (CNNs); Dermatology

### SS 2

### Assessing the impact of image quality on brain MRI diagnosis using deep learning

*Oge Marques, PhD[1]; Adam Ishay[1]; Ayah Elshaikh[1]; Whitney Angelica Andrews[1]; Anastasija Bulatovic[1]; and Huy Michael Do, MD[2]*

*[1] Florida Atlantic University*

*[2] National Institutes of Health (NIH) Clinical Center, Radiology and Imaging Sciences*

Short Summary: Image quality impairments in brain MRI are widely recognized as a problem that impacts both human experts and machine learning solutions. High-quality labeled data (with quality-related information) is costly, but deep learning solutions can assist in image evaluation by accounting for image quality. This work describes experiments designed to systematically test the following hypothesis: The performance of a Convolutional Neural Network (CNN)-based binary classifier for brain tumor detection decreases as the degree of image quality degradation on brain MRI images increases.

Purpose/Objectives: The purpose of this work is to demonstrate the impact of image quality on results produced by a deep learning algorithm for brain tumor detection.

Methods and materials: We used 253 images (155 tumorous, 98 non-tumorous) from Kaggle's *Brain MRI Images for Brain Tumor Detection* dataset. We processed the original images with different types of image quality degradation algorithms (Gaussian noise, Rician noise, motion blur) at varying degrees. The resulting images were then used as separate test datasets to evaluate the performance of a previously trained, fine-tuned, and validated CNN-based classifier (Fig. 1). Two architectures were used to test the performance for each dataset: ResNet-34 and AlexNet, both pretrained on ImageNet and fine-tuned for 20 and 35 epochs, respectively. The Adam optimizer was used with an initial learning rate of 0.001.

Results: We computed the accuracy for each combination of classifier and image quality level. For the baseline case, the accuracy of the CNN classifier was nearly 100%. The impact of Gaussian noise, Rician noise, and motion blur on the CNN classifier accuracy was proportional to the amount of blur/noise but affects the two architectures differently. ResNet was more robust to modest amounts of blur/noise than AlexNet for the same amount of quality loss.

Conclusion: We have empirically demonstrated that the impact of different types and degrees of image quality degradation on the accuracy of the results produced by a deep learning classification algorithm can be evaluated systematically, allowing us to derive precise conclusions about the dependence of the deep learning algorithm's performance on the quality of input pixel data from images.

Disclosure: Huy Do has a corporate research agreement with Carestream Health (Rochester, NY).
The other authors have no disclosures.

Keywords: Deep Learning; Convolutional Neural Networks (CNNs); Brain MRI; Image Quality

### SS 3

### Automated diagnosis of pneumothorax on chest radiographs using deep learning: *Understanding model predictions and limitations*

*L.J. Cornelissen, B.M. Pereira, V.M.A. Stirler, E.M. Fennema, P.M.A. van Ooijen; University Medical Center Groningen (UMCG), University of Groningen, The Netherlands*

Short Summary: We trained and evaluated a model for detection of pneumothorax on chest X-rays, using a publicly available dataset. While high classification performance was achieved, analysis of the model decision making revealed that the primary factor in the model decision making was the presence of an intercostal chest tube, making the model infeasible for clinical use.

Purpose/Objectives: To gain insight into model decision making and investigate the feasibility for clinical use of a deep learning model for detection of pneumothorax on chest X-rays, trained on a public dataset.

Methods and materials: A subset of 4388 images was extracted from the publicly available dataset ChestXray14. Half of the radiographs exhibits a pneumothorax, the other half does not. This dataset was split into a train, validation and test set (80/10/10 percent of the data, respectively). The training and validation sets were used as input for training several classification models. Models based on two different architectures were compared (InceptionV3 and ChexNet), and the test set was used to evaluate model performance. Standard data augmentation methods (random rotation, scaling and translation) were used to enlarge the training set.

Several visualization methods were then applied to generate saliency maps for the images classified by the models, in order to shed light on the model decision making. A saliency map highlights those regions in the image that contribute most to the final classification result of the model, and can therefore be used to gain insight in why a model predicts a particular label for a certain image.

Results: On the test set, we obtained an area under the roc curve of 0.86 (0.83) and a maximum F1 score of 0.8 (0.81) for the model based on InceptionV3 (ChexNet), indicating reasonable classification performance. However, the saliency maps and error analysis of the classifier revealed that model attention was focused on intercostal chest tubes, used in the treatment of a pneumothorax.

Conclusion: While an accurate classification model for pneumothorax can be trained based on the ChestXray14 dataset, the high prevalence of post-treatment images in the set results in a bias towards images with tubes, which is problematic for clinical use.

Disclosure: L.J. Cornelissen is also affiliated to COSMONiO Imaging Ltd.

Keywords: Thorax radiographs, deep learning, pneumothorax

### SS 4

### Dynamic Contrast-Enhanced Perfusion MRI and Diffusion-Weighted Imaging as an imaging biomarker for paediatric cancer

*Leonor Cerdá Alberich, Gracia Martí Besa, Ángel Alberich-Bayarri, Luis Martí-Bonmatí*

Short Summary: The usefulness of MR imaging in children with neuroblastic tumors was examined by using quantitative parameters derived from diffusion-weighted and dynamic contrast-enhanced MRI.

Histogram analysis and machine learning algorithms were used to estimate reproducible and meaningful imaging biomarkers, and pseudoexperiments were utilised to identify and remove the noise originating from low-quality voxels.

The proposed novel methodology was observed to successfully differentiate benign and malignant neuroblastic tumors in terms of the apparent diffusion coefficient and the initial area under the curve at 60 seconds, and precise uncertainties were assigned to these quantities. This work lays the foundation for future advances in reproducible imaging biomarkers for paediatric solid tumor cancers.

Purpose/Objectives: Neuroblastoma (NB) is the most frequent solid extracranial cancer in childhood. Its diagnosis, prognosis and monitoring are based on the information provided by multiparametric magnetic resonance (MR) and MIBG images. Our focus in this work is to explore the utility of diffusion and perfusion changes in NB as an early biomarker of diagnosis, using diffusion-weighted (DWI) and dynamic contrast-enhanced (DCE) MRI.

Methods and materials: Multiple MR imaging real-word sequences, available within the H2020 PRIMAGE project, were used from 30 patients. Volumes-of-interest were calculated and transferred to DCE perfusion and apparent diffusion coefficient (ADC) maps. Histogram analysis and clustering based unsupervised machine learning algorithms were used to determine the values of mean and standard deviation of initial area under the curve at 60 seconds (IAUC60) and ADC for automatic differentiation of neuroblastic tumors. The resolution of voxel intensity was estimated and the IAUC60 and ADC data was smeared accordingly to allow us to identify and remove the noise and low-quality voxels (defined as those voxels with an uncertainty larger than 10%).

Results: Significant differences in mean ADC (expressed in $10^{-3}\,\mathrm{mm}^2/s$) were found for neuroblastic tumors: 1.2 for ganglioneuroma, 0.74 for ganglioneuroblastoma, and 0.54 for neuroblastoma, with an uncertainty of 37%, 43% and 20%, respectively. This result improves tumor differentiation with respect to state-of-the-art voxel-by-voxel methodologies, which were found to be: 1.6 for ganglioneuroma, 1.7 for ganglioneuroblastoma, and 1.3 for neuroblastoma, with an uncertainty of 45%, 63% and 23%, respectively. Mean IAUC60 was found to have a value of 43 (and 14% uncertainty) for neuroblastoma, as opposed to a value of 17 (and 17% uncertainty) with state-of-the-art voxel-by-voxel methodologies.

Conclusion: The proposed novel technique to determine IAUC60 and ADC parameters holds promise for differentiating benign and malignant neuroblastic tumors.

Disclosure: Nothing to disclose.

Keywords: Diffusion-weighted imaging; Dynamic contrast-enhanced; Paediatric cancer; Imaging biomarker; MRI

### SS 5

### Pre-post exercise evaluation of femoral cartilage thickness and T2 changes in ultramarathon athletes

*Fabio García-Castro[1], Jordi Catalá March[2], Ana Jiménez-Pastor[1], Daniel Brotons Cuixat[3], Miquel Llobet Llambrich[3], Eduard Sánchez Osorio[3], Ángel Alberich-Bayarri[1]*

1. *QUIBIM S.L., Valencia, Spain*
2. *Instituts Guirado, Barcelona, Spain*
3. *Centro Ergodinámica Clínica, Barcelona, Spain*

Short Summary: T2-mapping is a useful imaging biomarker to evaluate the integrity of the femoral cartilage matrix after a high level exercise (ultramarathon). Significant differences were found in T2 relaxation times pre and post exercise. However, these differences disappeared in knees injected with hyaluronic acid. No significant differences were found in cartilage thickness.

Purpose/Objectives: Quantification of T2 relaxation times and thickness of articular cartilage in MRI provides information of the integrity and condition of the tissue matrix. The assessment of changes in these imaging biomarkers pre and post exercise is the main objective of this work.

Methods and materials: Both knees of 9 asymptomatic healthy subjects were acquired before and after an 80 km run (ultramarathon), obtaining a total of 36 MRI studies. A T2 multi-echo sequence with 6 echo times and a high-resolution anatomical sequence (DESS) were included in the MRI protocol. An elastic registration algorithm was used on the multi-echo T2 sequence, using the anatomical sequence as reference, to achieve spatial consistency. An in-house algorithm that implements the distance transform was used to calculate the thickness of the femoral cartilage at each point of the cartilage surface. Signal decay for each voxel on the multi-echo T2 sequence was modelled using a non-linear least-squares fit algorithm, obtaining the T2 mapping of the femoral cartilage. Thanks to the previous registration, T2 and thickness parametric maps were aligned to the same spatial reference. Thickness and T2 values were statistically compared before and after the 80 km run, as well as with and without the administration of hyaluronic acid.

Results: Significant differences were obtained in the median ($p=0.037$) and 25th percentile ($p=0.017$) of T2 values before and after exercising (higher post-ultramarathon values). In cases where hyaluronic acid was injected, these differences disappeared ($p=0.24$ and 0.16). There were no significant differences in cartilage thickness.

Conclusion: As demonstrated in the results section, T2-mapping provides information on post-ultramarathon alterations. In knees where hyaluronic acid was injected, there were no significant differences in T2 values. Femoral cartilage thickness remained unchanged during the exercise.

Disclosure: Nothing to disclose

Keywords: T2 mapping, imaging biomarkers, sports medicine, radiology, MRI, quantification

### SS 6

### Automated Lung Segmentation in Chest Radiographs using Deeply Supervised Convolutional Neural Networks Trained by means of a Database Augmented with a Generative Adversarial Neural Network.

*Rafael López-González, Mar Roca-Sogorb, Fabio García-Castro, Ángel Alberich-Bayarri*

Short Summary: The study describes an Artificial Intelligence methodology that is able to automate the segmentation of lungs in chest radiographs by means of Convolutional Neural Networks (CNN) and Generative Adversarial Neural Networks (GAN) achieving a Dice Score of 0.94.

Purpose/Objectives: Automated lung segmentation in Chest X-rays can bring many benefits for the optimization of image analysis pipelines, such as lung textures quantification and pathologies classification, which in turn will help to speed up the radiological workflow and to foster new discoveries with radiomic studies. Our goal was to improve lung segmentation by using virtually augmented cases using GAN.

Methods and materials: Three public datasets of chest radiographs with manual segmentation masks of the lungs were gathered for the purpose of training a Deep Learning model. The Montgomery database with 138 studies, the Shenzen database with 662 studies and the JRSC database with 247 studies. Additionally, a 100 manually segmented cases from the ChestXray14 database were used for testing purposes.

The training databases contained radiographs with tuberculosis, nodules and healthy subjects. To increase the variability of the dataset, the radiographs from healthy subjects of the Montgomery and JSRT databases were augmented by means of a GAN that was trained to introduce realistic pathologies into chest radiographs. With this approach the size of the database was increased to a total of 3343 images.

To evaluate the influence of this advanced data augmentation, a deeply-supervised UNET was trained with and without the augmented samples. The performance of both models was measured by means of the Dice Score coefficient in between the predictions of both models and the manual segmentations of the ChestXray14 database.

Results: The model trained without the augmented samples obtained an average Dice score of 0.86 for the right lung and 0.91 for the left lung, with 0.97 for the background label. The model trained with the augmented samples obtained an average Dice score of 0.94 for the right lung, and 0.95 for the left lung, with 0.98 for the background label.

Conclusion: Optimizing the segmentation of the lungs in chest radiographs is feasible by means of Convolutional Neural Networks trained with augmented data. The use of these techniques allows to learn robust segmentation models and refine them with the generation of realistic augmented data.

Disclosure: No conflicts of interest to disclose

Keywords: Convolutional Neural Networks, Artificial Intelligence, Lung Segmentation, Chest Radiographs

### SS 7

### Image analysis using an intercontinental infrastructure for the deployment of trustworthy cloud services: the ATMOSPHERE project

*Ignacio Blanquer (Universitat Politècnica de València), Eduardo Camacho-Ramos (QUIBIM S.L.), Andrey Brito (Universidade Federal de Campina Grande), Ana Jimenez-Pastor (QUIBIM S.L.), Christof Fetzer (TU Dresden), Altigran da Silva (Universidade Federal do Amazonas), Andre Carvalho (Universidade Federal do Amazonas), Amanda Calatrava (Universitat Politècnica de València), Fabio García-Castro (QUIBIM S.L.), Ángel Alberich-Bayarri (QUIBIM S.L.), Francisco Brasileiro (Universidade Federal de Campina Grande)*

Short Summary: The ATMOSPHERE platform provides services for the development, management and trustworthy assessment of distributed Artificial Intelligence applications from different cloud providers. An intercontinental infrastructure across Europe and Brazil in the frame of medical imaging processing is herein presented.

Purpose/Objectives: To design and implement a federated cloud platform for Data Analytics capable of dealing with sensitive medical data, especially medical imaging. To measure the trustworthiness of the applications and services on the platform.

Methods and materials: The application relies on a back-end framework that orchestrates the execution of containers on a cloud federation powered by the Fogbow middleware. This application is based on an Elastic Kubernetes cluster that manages containers and services; a CLUES manager linked to Kubernetes and the deployment service; and a persistent storage service. All processing is done using Vallum, which is an access, authorization and privacy management module for sensitive data, aiming to prevent its vulnerability. All sensitive data is processed using SGX containers, which protects them from memory reading-based attacks. It also includes an anonymization module responsible for removing potentially identifying information from medical images. Once critically sensitive information has been securely removed, images can leave the infrastructure deployed in Brazil and be stored on the European volumes. The GPU resources available in Europe are leveraged to perform the data processing. This infrastructure was used to set up a secure virtual environment to create an automatic classifier of echo-cardio images for the screening of Rheumatic Heart Disease (RHD). The application employed high-end computing resources and ensured that sensitive data were only accessible within the region of origin.

Results: This architecture provided a secure environment to store and process 5600 echo-cardio exams as of today with the utmost trustworthiness and security to rule out RHD.

Conclusion: The ATMOSPHERE platform has proven to offer a secure framework for deploying radiological imaging applications able to keep critical data encrypted and making them accessible only in trusted execution environments.

Disclosure: The work in this abstract is mainly developed in the context of the project ATMOSPHERE, funded jointly by the European Commission under the Cooperation Programme, Horizon 2020 grant agreement No 777154 and the Brazilian Ministério de Ciência, Tecnologia e Inovação (MCTI), number 51119

Keywords: Cloud Computing, Trustworthiness, Privacy.

### SS 8

### Deep Learning as a Service

*Lara Lloret Iglesias (Instituto de Física de Cantabria (UC-CSIC)), Keiichi Ito (Helmholtz Zentrum München), Wolfgang zu Castell (Helmholtz Zentrum München)*

Short Summary: Deep Learning (DL) is nowadays at the forefront of Artificial Intelligence, shaping tools that are being used to achieve very high levels of accuracy in many different research fields such as health care in general and medical imaging in particular. The DEEP as a Service (DEEPaaS) approach developed within the DEEP-HybridDataCloud project (https://deep-hybrid-datacloud.eu/) allows to train, serve and share a DL model in a user friendly way, lowing the entry barrier for non-experts.

Purpose/Objectives: Training a Deep Learning model is a very complex and computationally intensive task requiring the user to have a full setup involving a certain hardware, the adequate drivers, dedicated software and enough memory and storage resources. Very often the DL final user is not a computing expert, and want all of this technology as accessible and transparent as possible to be able to just focus on creating a new model or applying a pre-built one to some data. Besides, the sharing of the trained model usually requires the final user to have enough knowledge to be able to use it. The objective of the DEEP-HybridDataCloud project is to develop an open framework for all users, and not just for a few experts, enabling the transparent training, sharing and serving of Deep Learning models both locally or on a cloud system.

Methods and materials: The user doesn't need to worry about compatibility problems since everything has already been tested and encapsulated allowing to have a fully working model in just a few minutes. The technology used is based on Docker containers that pack already all the tools needed to deploy and run the DL models in the most transparent way.

Results: In the DEEP Open Catalog (https://marketplace.deep-hybrid-datacloud.eu/) you can find ready to use modules in a variety of domains, including a use case on diabetic retinopathy that automatically classifies biomedical images of the retina into five disease categories or stages (from healthy to severe). These modules can be executed on your local laptop, on a production server or on a cloud system. To make things even easier, we have developed an API allowing the user to interact with the model directly from the web browser. It is possible to perform inference, train or check the model metadata just with a simple click.

Conclusion: The DEEP as a Service (DEEPaaS) is a transparent solution allowing non-experts to fully exploit the potential of the most cutting edge Deep Learning techniques for data analysis.

Disclosure: This software has been developed within the DEEP-Hybrid-DataCloud (Designing and Enabling E-infrastructures for intensive Processing in a Hybrid DataCloud) project that has received funding from the European Union's Horizon 2020 research and innovation programme under grant agreement No 777435.

Keywords: deep learning, machine learning, artificial intelligence, cloud, transparent approach, user friendly, deepaas

### SS 9

### What kind of legal and ethical issues will arise from using AI systems in the medical practice?

*P. Menéndez Fernández-Miranda, P. Sanz Bellón, A. Pérez del Barrio, D. Rodríguez González, L. Lloret Iglesias, J.A. Vega Álvarez.*

Short Summary: The arrival of AI to medicine is imminent, and it is not coming alone. Such a huge revolution necessarily implies new ethical and legal challenges that humans have never faced before. Solving these issues will be crucial to determine the future of Artificial Intelligence (AI) techniques in medicine in the following years.

Purpose/Objectives: To exhibit the legal and ethical issues related to the use of AI-based technologies in medicine.

Methods and materials: A systematic review of all articles on ethical and legal issues related to AI in Medicine published in PubMed-indexed journals, was carried out. No language or publication date restrictions, were imposed.

Results: Two sources of ethical and legal conflicts were found. The first source arises from the AI systems building process. AI programs need a certain amount of clinical data usually belonging to the patient's private life, and revealing or using this data may interfere in medical confidentiality. Regarding that issue, it is mandatory to establish the ethical principles and the legal rules that must regulate the sharing of patient´s data. Bio-banks of medical data could be a great opportunity to create an ethical and a legal framework.

The other important challenge concerns the use of AI systems in the medical practice. AI software is designed to help radiologists in making decisions, so AI programs will have an influence on the patient's outcome. It means that AI systems will take a big part in clinical decisions, such as conclude a diagnosis or decide the best treatment. Hence, the point is, who is the responsible for these decisions in the eyes of the law? What kind of ethical issues will arise from using a non-moral system in medicine?.

Consequently, in order to find out the answers to these questions, the four basic principles of medical ethics have to be considered: beneficence, non-maleficence, justice and autonomy.

Conclusions: The ethical and legal issues related to the implantation of AI systems in the medical practice will demand a multidisciplinary approach with help from multiple professionals, such as doctors, engineers, politicians and jurists. It will be vital to train doctors in AI because their insight will be essential for the development of solid AI medical systems.

Disclosure: Nothing to disclose.

Keywords: artificial intelligence, ethics, legality.

### SS 10

### Is radiology ready for the implementation of artificial intelligence? An international survey of 1,041 radiologists and residents

*Merel Huisman, MD PhD; Erik Ranschaert, MD, PhD; William Parker, MD, BMSc; Domenico Mastrodicasa, MD; Martin Koci, MD; Daniel Pinto de Santos, MD; Francesca Coppola, MD; Sergey Morozov, MD PhD MPH; Marc Zins, MD; Cedric Bohyn, MD; Curtis Langlotz, MD PhD; Nicola H Strickland, MD ; Ural Koç, MD[12]; Shengwen Yang, MD[4]; Jie Wu, MS[13]; Satyam Veean, MD[14]; Dominik Fleischmann, MD[4]; Tim Leiner, MD, PhD[15]; Martin J Willemink, MD PhD[4]*

Short Summary: In this survey a small majority of residents and radiologists expects no negative effects on the labor market, and almost half has an open and pro-active attitude towards AI. This attitude can be augmented by having AI-specific knowledge, indicating the importance of additional training to facilitate implementation.

Purpose/Objectives: Key opinion leaders in AI (artificial intelligence) claim that radiologists should drive the next phase. However, the perception at large by radiologists and residents of AI remains unexplored. We sought to investigate the knowledge and overall attitude towards AI in radiologists and residents.

Methods and materials: A web-based multi-language survey was accessible between April-July 2019. The survey consisted of 39 questions on knowledge and attitude towards AI. Multivariable logistic regression was used identify independent predictors for an open and proactive attitude towards AI (willingness to use and learn about AI, to collaborate with data scientists, and agreement that radiologists should take a leadership role in AI).

Results: A total of 1,041 respondents with a mean age of 41 (range 24-74) from 54 countries completed the survey. Most participants were male (n=670, 65%), radiologist (n=719, 69%), worked in non-academic centers (n=572, 55%) without scientific background (n=727, 70%). Almost half had some knowledge of informatics/statistics (n=504, 48%). The majority had at least basic AI-specific knowledge (n=771, 74%). More than half had no fear of replacement (n=640, 61%). Almost half of participants demonstrated an open and proactive attitude towards AI (n=501, 48%). The strongest significant ($p<0.001$) predictors for an open and proactive attitude were AI-specific knowledge (OR 2.32, CI 1.67-3.23) and male gender (OR 1.94, CI 1.45-2.59) independent of age, region and current position.

Conclusions: This international survey indicated that almost half of the respondents had an open and proactive attitude towards AI, and more than half did not expect negative effects on the labor market. Any level of AI-specific knowledge was the strongest predictor for having an open and proactive attitude, indicating the need for additional training on AI in residents and radiologists.

Disclosure: Nothing do disclose

Keywords: Artificial Intelligence, survey, education, policy
